# Supplementary material for: Endothelial cell-derived stem cell factor promotes lipid accumulation through c-Kit-mediated increase of lipogenic enzymes in brown adipocytes
Source: Nat Commun. 2023 May 13;14:2754. doi: 10.1038/s41467-023-38433-5 (PMC10183046; doi:10.1038/s41467-023-38433-5)
Supplement: Supplementary file 1 — Supplementary Information [file 41467_2023_38433_MOESM1_ESM.pdf]

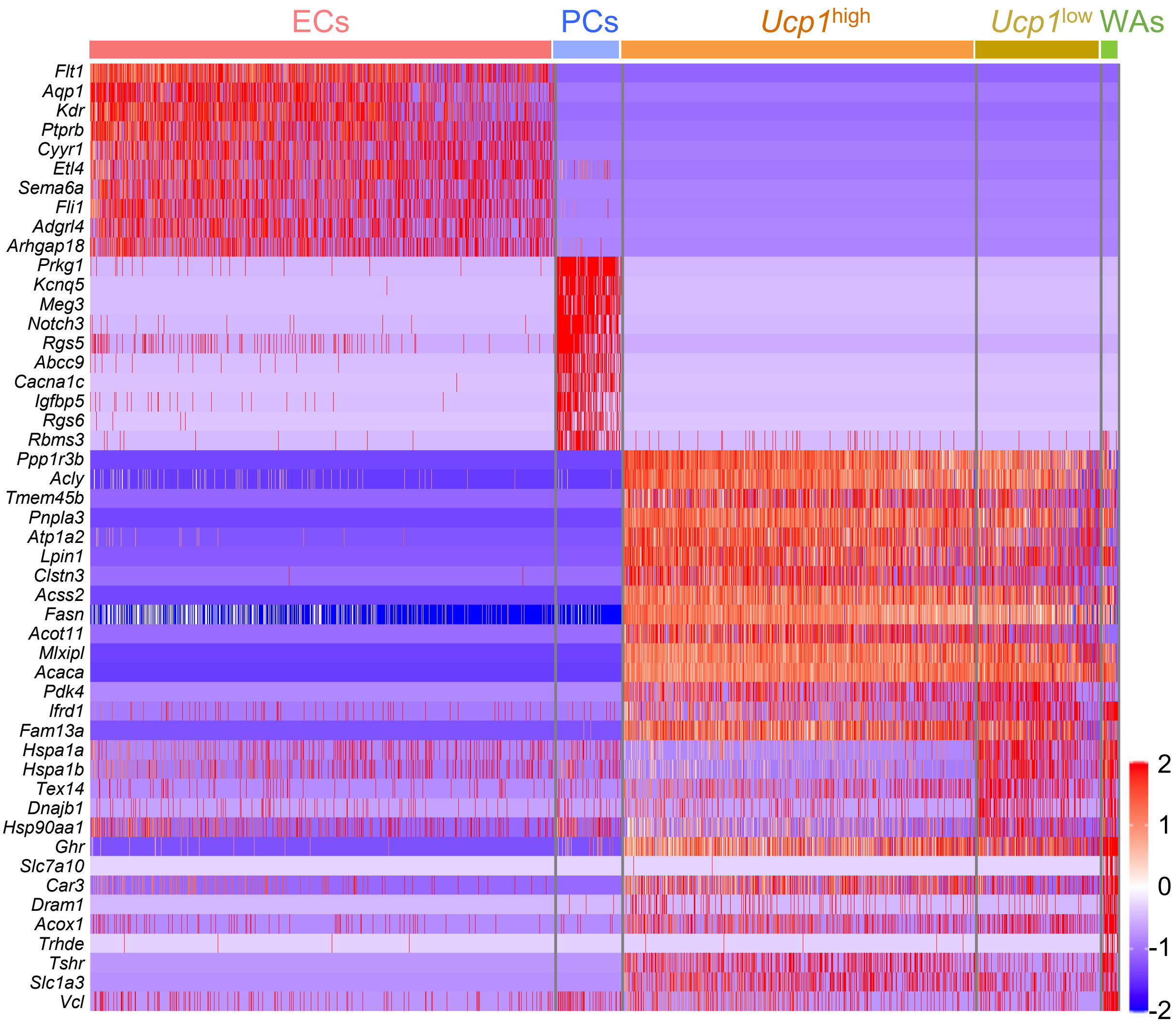

Supplementary Fig. 1. Heatmap showing differentially expressed genes in ECs, PCs, *Ucp1*^{high} BAs or *Ucp1*^{low} BAs, and WAs in the BAT of adult C57BL/6J mice.

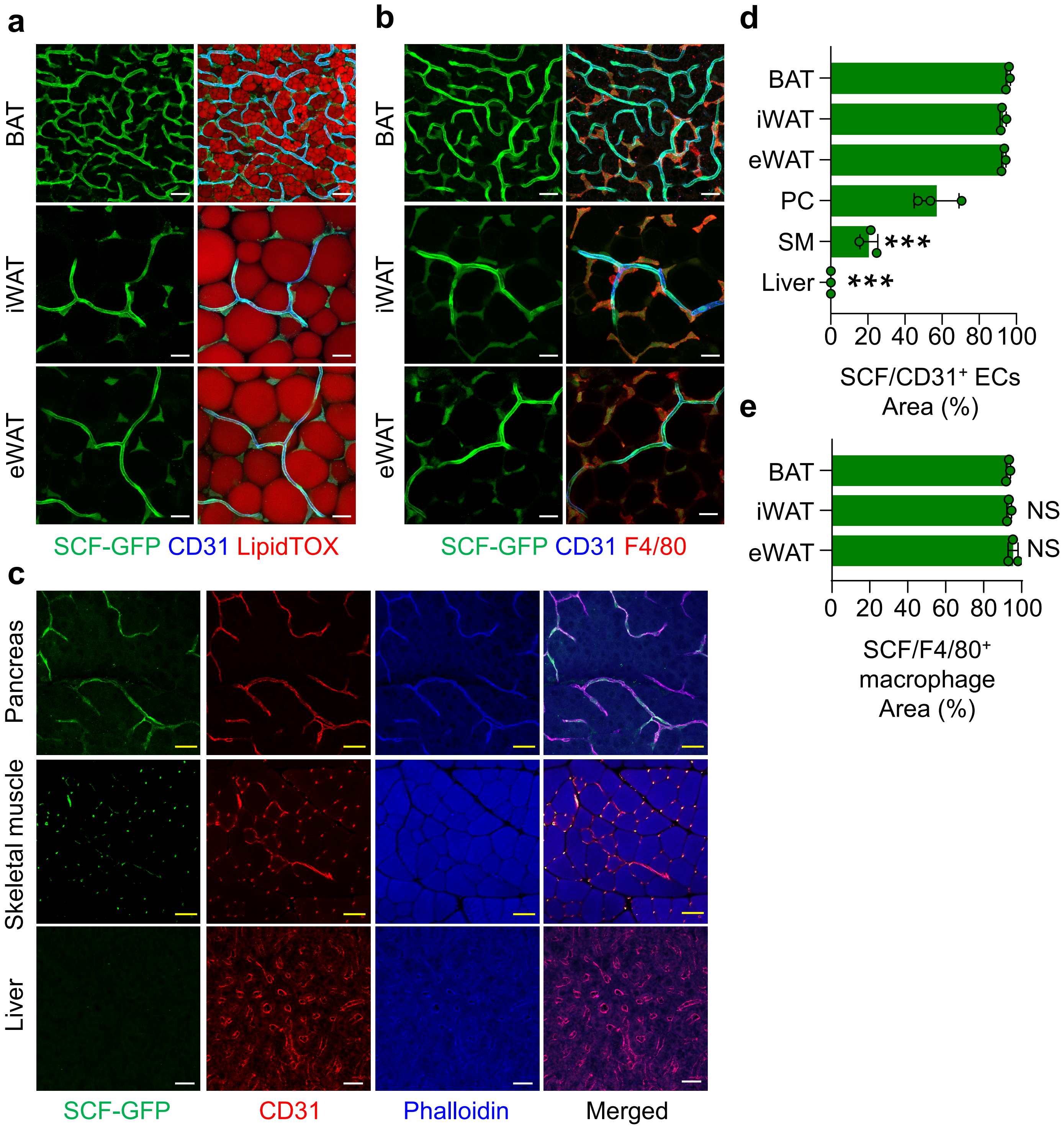

Supplementary Fig. 2. Lineage tracing of SCF in major metabolic organs

a-e, Representative images and comparisons showing SCF expression in CD31⁺ ECs and F4/80⁺ macrophages but not in LipidTOX⁺ adipocytes in the indicated organs of 8-week-old *SCF*-GFP reporter mice. Phalloidin⁺ cells indicate each parenchymal cell of indicated organs. Scale bars, 20 μm (white) and 100 μm (yellow). BAT, brown adipose tissue; iWAT, inguinal white adipose tissue; eWAT, epididymal WAT; PC, pancreas; SM, skeletal muscle. Each dot indicates a value from one mouse and n = 3 mice/group from two independent experiments. Horizontal bars indicate mean ± SD. ***P<0.001 *versus* BAT by one-way ANOVA test followed by Tukey's *post-hoc* test. NS, not significant.

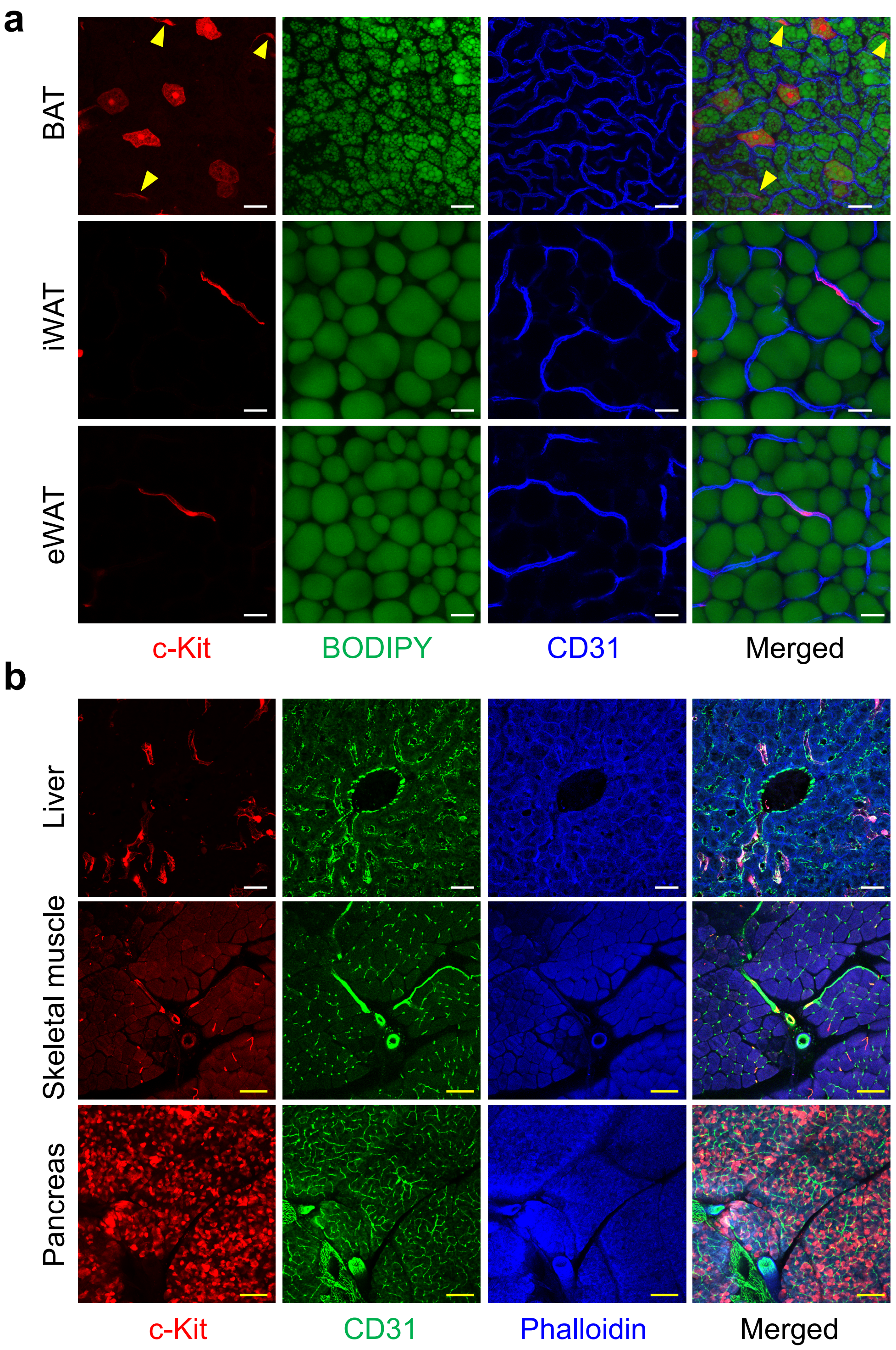

Supplementary Fig. 3. Lineage tracing of c-Kit in major metabolic organs
a,**b**, Representative images showing c-Kit expression in CD31^{+} ECs in BAT (yellow arrowheads), WATs, liver and skeletal muscle, and BODIPY^{+} BAs in BAT but not in BODIPY^{+} white adipocytes in WATs of 8-week-old *c-Kit*^{iTR} mice. Phalloidin^{+} cell indicates each parenchymal cell of indicated organs. Similar findings were obtained from n = 3 mice/group from two independent experiments. Scale bars, 20 μm (white) and 100 μm (yellow).

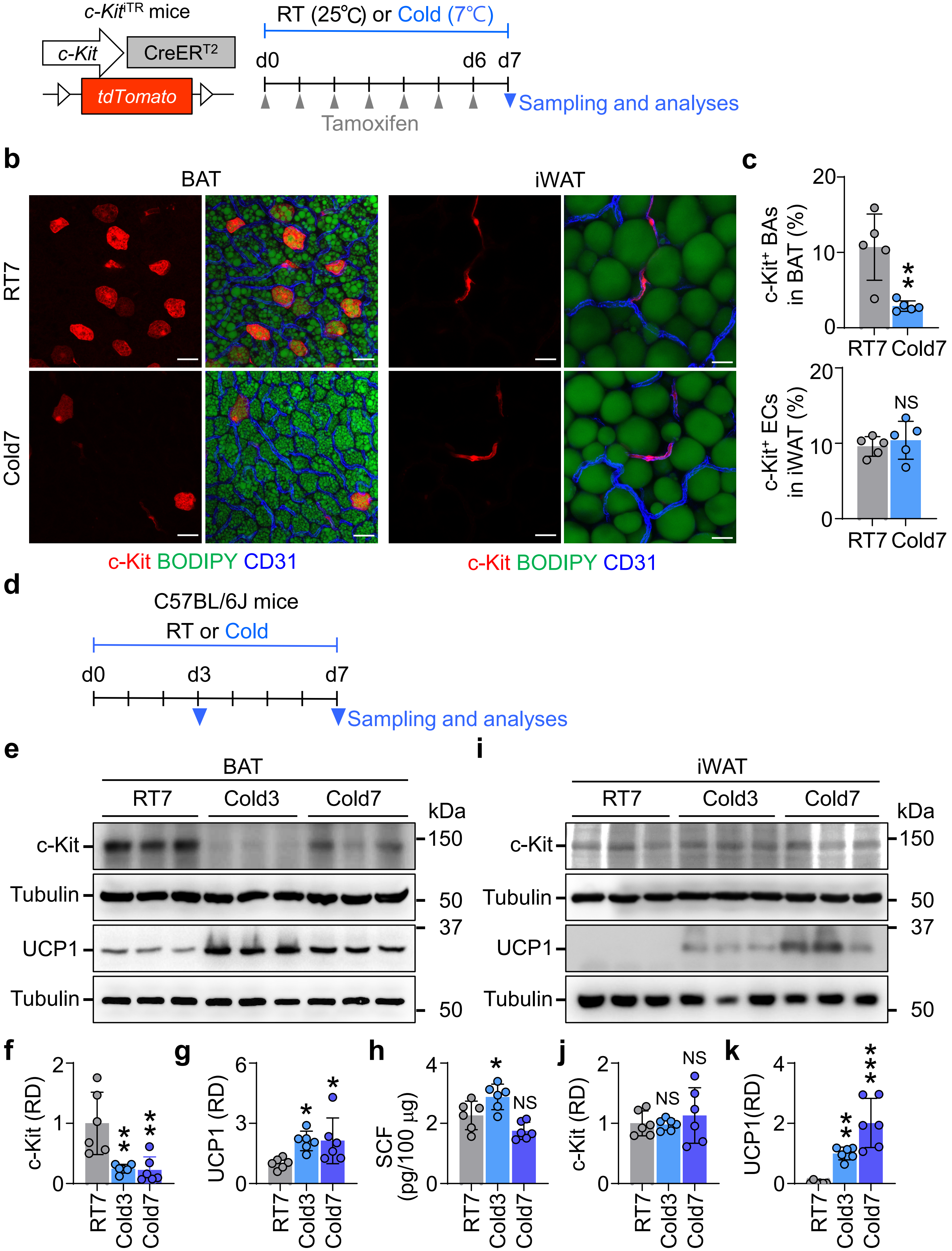

a
c-Kit^iTR mice
c-Kit
CreER^T2
tdTomato
c-Kit^iTR mice
RT (25℃) or Cold (7℃)
d0
d6
d7
Sampling and analyses
Tamoxifen
b
BAT
iWAT
RT7
Cold7
c-Kit BODIPY CD31
c-Kit BODIPY CD31
c
c-Kit⁺ BAs in BAT (%)
c-Kit⁺ ECs in iWAT (%)
RT7
Cold7
NS
d
C57BL/6J mice
RT or Cold
d0
d3
d7
Sampling and analyses
e
BAT
RT7
Cold3
Cold7
kDa
c-Kit
Tubulin
UCP1
Tubulin
150
50
37
50
i
iWAT
RT7
Cold3
Cold7
kDa
c-Kit
Tubulin
UCP1
Tubulin
150
50
37
50
f
c-Kit (RD)
g
UCP1 (RD)
h
SCF (pg/100 μg)
NS
j
c-Kit (RD)
NS
NS
k
UCP1(RD)
RT7
Cold3
Cold7

Supplementary Fig. 4. Cold exposure reduces the number of c-Kit⁺ BAs and protein level of c-Kit in BAT but not in iWAT

a, Diagram depicting the generation of c-Kit reporter (*c-Kit*^{iTR}) mouse, and the exposure of 8-week-old *c-Kit*^{iTR} mice to room temperature (RT, 25℃) or cold (Cold, 7℃) for 7 days (RT7 or Cold7) with tamoxifen administrations.
b,**c**, Representative images and comparison of the number of c-Kit^{+} BAs in BATs or the area of c-Kit^{+} ECs to total area of CD31^{+} ECs in iWATs from *c-Kit*^{iTR} mice at RT7 or Cold7. Scale bars, 20 μm. Each dot indicates a value from one mouse and n = 5 mice/group from two independent experiments. Vertical bars indicate mean ± SD. ***P*<0.01 *versus* RT7 by two-tailed t-test. NS, not significant.
d, Diagram depicting the experimental procedure for the exposure of 8-week-old C57BL/6J mice to RT or cold for 3 or 7 days (Cold3, RT7, or Cold7).
e-**k**, Representative immunoblotting and comparisons of relative density (RD) of c-Kit and UCP1 in BAT or iWAT of C57BL/6J mice at RT7, Cold3, and Cold7. The same amount of protein loading in each lane is verified by immunoblotting of tubulin. Comparisons of SCF concentration in BAT at RT7, Cold3, and Cold7 (**h**). Each dot indicates a value from one mouse and n = 6 mice/group from two independent experiments. Vertical bars indicate mean ± SD. **P*<0.05, ***P*<0.01, and ****P*<0.001 *versus* R7 by one-way ANOVA test followed by Tukey’s *post-hoc* test. NS, not significant. Protein sizes are indicated as kilodalton (kDa).

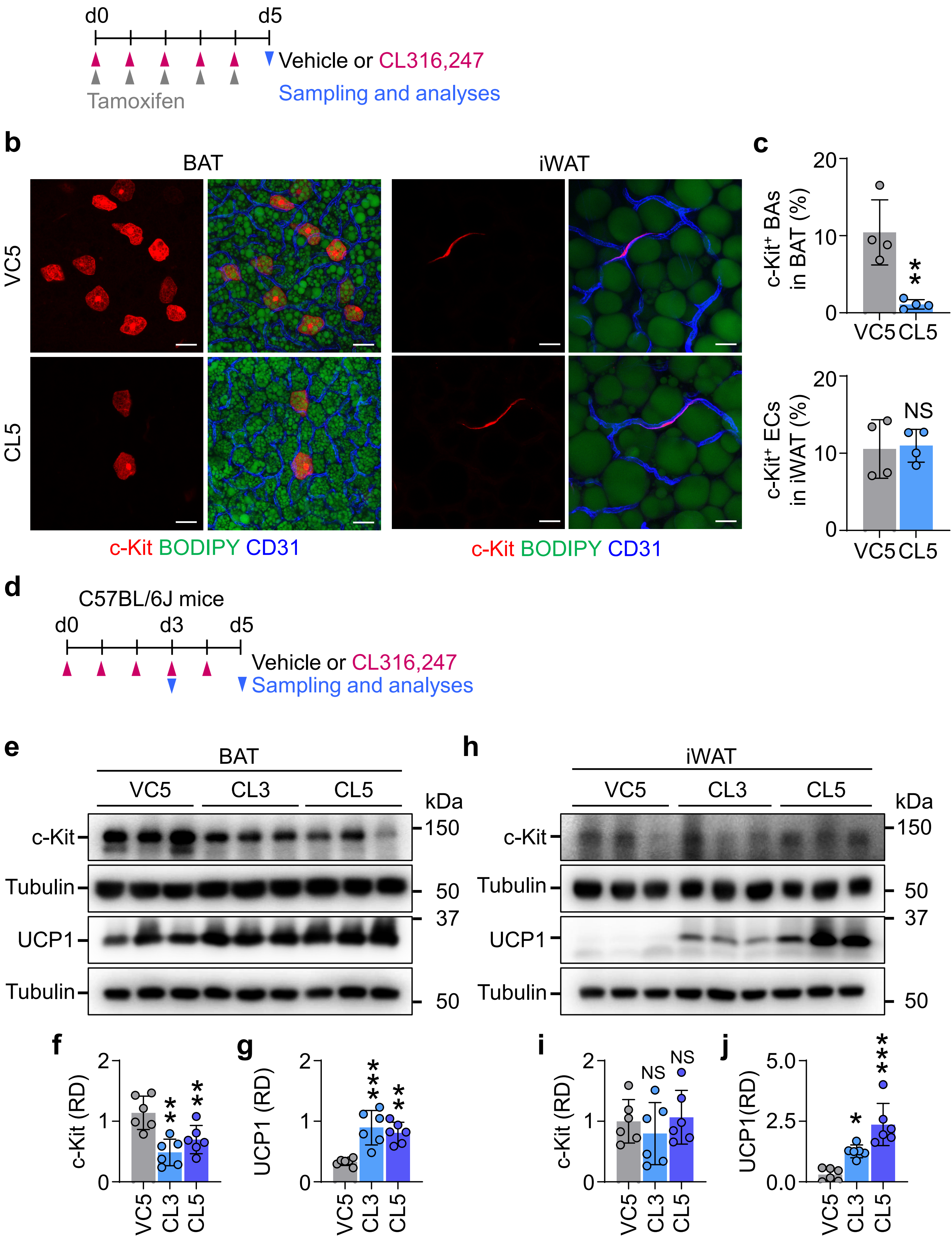

a
c-Kit^iTR mice
d0
d5
Vehicle or CL316,247
Tamoxifen
Sampling and analyses
b
BAT
iWAT
VC5
CL5
c-Kit BODIPY CD31
c-Kit BODIPY CD31
c
c-Kit+ BAs in BAT (%)
**
c-Kit+ ECs in iWAT (%)
NS
VC5 CL5
d
C57BL/6J mice
d0
d3
d5
Vehicle or CL316,247
Sampling and analyses
e
BAT
VC5 CL3 CL5
kDa
c-Kit
Tubulin
UCP1
150
50
37
h
iWAT
f
c-Kit (RD)
g
UCP1 (RD)
i
j
UCP1(RD)
NS
*
**
***

Supplementary Fig. 5. β3-AR activation reduces the number of c-Kit$^+$ BAs and protein level of c-Kit in BAT

a, Diagram depicting the experimental procedure for intraperitoneally (i.p.) administration of vehicle or CL316,247 with simultaneous tamoxifen administrations for 5 consecutive days (VC5 or CL5) to 8-week-old *c-Kit*^{iTR} mice.
b,**c**, Representative images and comparison of the number of c-Kit$^+$ BAs in BATs or CD31$^+$ ECs in the iWATs from *c-Kit*^{iTR} mice after VC5 and CL5. Scale bars, 20 μm. Each dot indicates a value from one mouse and n = 4 mice/group from two independent experiments. Vertical bars indicate mean ± SD. ***P*<0.01 *versus* VC5 by two-tailed t-test.
d, Diagram depicting the experimental procedure for daily i.p. administration of vehicle (VC5) or CL316,247 for 3 or 5 consecutive days (CL3 or CL5) to 8-week- old C57BL/6J mice.
e-**j**, Representative immunoblotting and comparisons of relative density (RD) of c-Kit and UCP1 in BAT or iWAT at VC5, CL3, and CL5. The same amount of protein loading in each lane is verified by immunoblotting of tubulin. Each dot indicates a value from one mouse and n = 6 mice/group from two independent experiments. Vertical bars indicate mean ± SD. **P*<0.05, ***P*<0.01, and ****P*<0.001 *versus* VC5 by one-way ANOVA test followed by Tukey's *post-hoc* test. NS, not significant. Protein sizes are indicated as kilodalton (kDa).

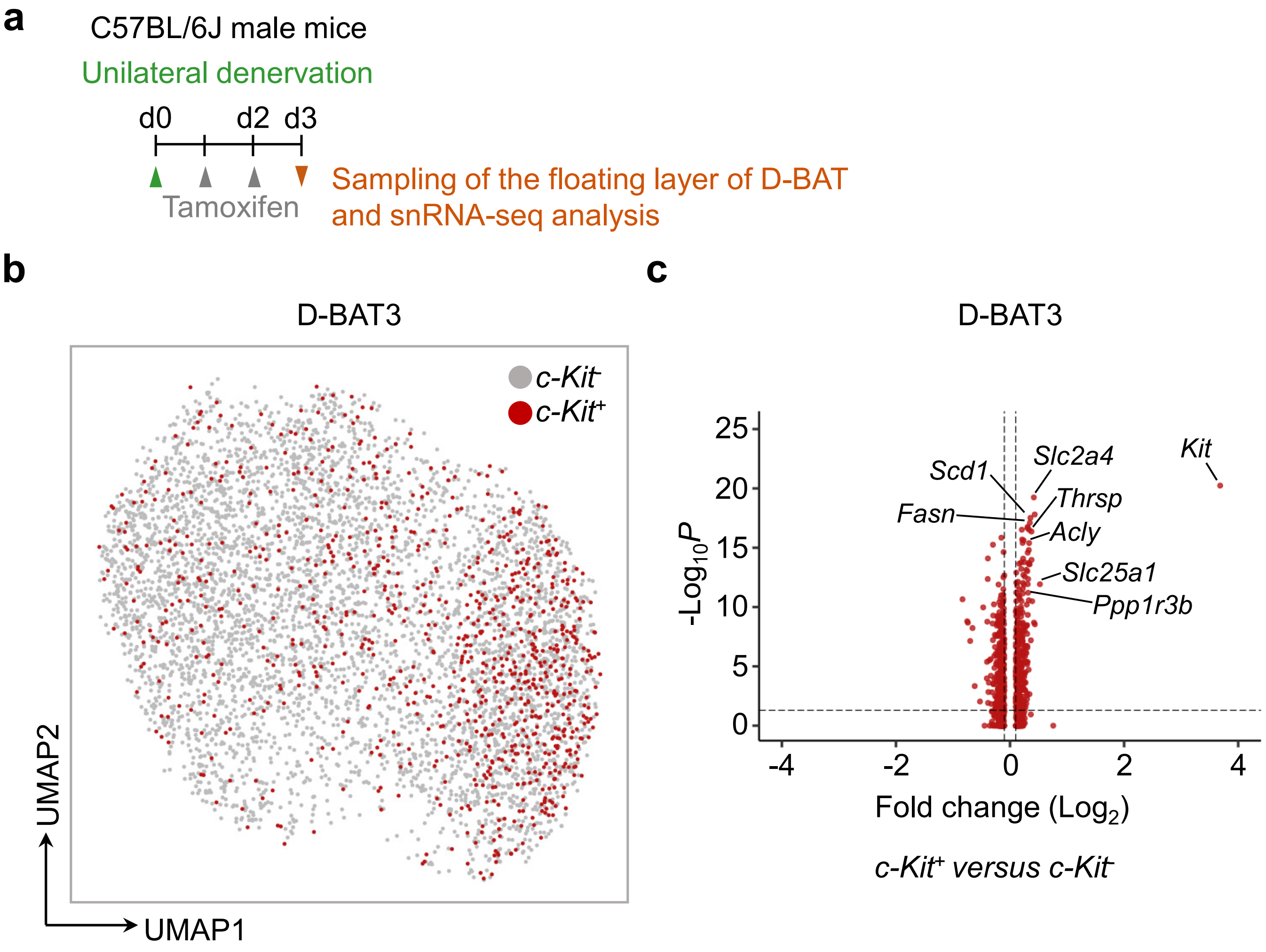

Supplementary Fig. 6. The gene expressions related to lipogenesis are upregulated in *c-Kit*$^{+}$ BAs.

a, Diagram depicting the experimental procedure for unilateral denervation in the interscapular BAT of 8-week-old C57BL/6J male mice (n = 20), tamoxifen administrations, and sampling for snRNA-seq at 3 days later (D-BAT3) for the analyses.
b, UMAP plots showing the distribution of *c-Kit*$^{+}$ or *c-Kit*$^{-}$ BAs in the D-BAT3.
C, Volcano plot showing the gene whose significance is P<0.05 in *c-Kit*$^{+}$ *versus c-Kit*$^{-}$ BAs in the D-BAT3. For differential expression testing, we implemented MAST(https://genomebiology.biomedcentral.com/articles/10.1186/s13059-015-0844-5) and p-values were adjusted by Bonferroni method. Interested genes are highlighted with indicating gene names.

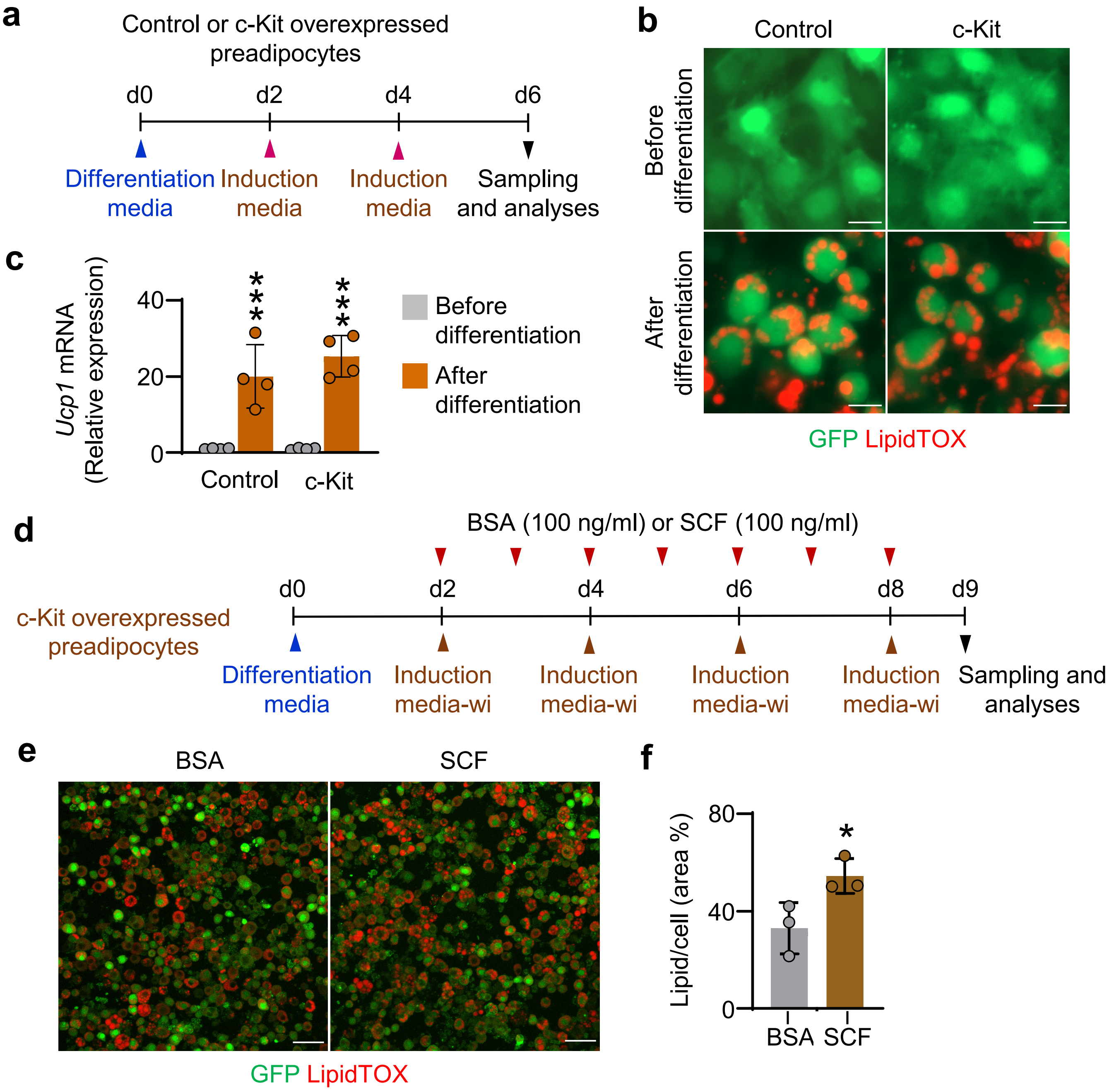

Supplementary Fig. 7. SCF enhances lipid accumulation in cultured BAs

a, Diagram depicting the experimental procedure for differentiation of Control (transfected with empty lentiviral plasmid) or c-Kit overexpressed (transfected with *c-Kit* cDNA inserted lentiviral plasmid; c-Kit) in preadipocytes.

b,**c**, Representative images of LipidTOX staining and relative expression of *Ucp1* mRNA before and after differentiation in the adipocytes of Control and c-Kit. Scale bars, 20 μm. Dots and bars indicate mean ± SD from n = 4/group from two independent experiments. ****P*<0.001 *versus* before differentiation by two-tailed t-test.

d, Diagram depicting the experimental procedure for differentiation of c-Kit overexpressed preadipocytes with the induction media without insulin (-wi), and daily treatment of BSA (100 ng/ml) or SCF (100 ng/ml).

e,**f**, Representative images and comparison of LipidTOX-stained lipid accumulation treated with BSA or SCF for 7 days on the c-Kit over-expressed BAs. Scale bars, 50 μm. Dots and bars indicate mean ± SD from n = 3/group from two independent experiments. **P*<0.05 *versus* BSA by two-tailed t-test.

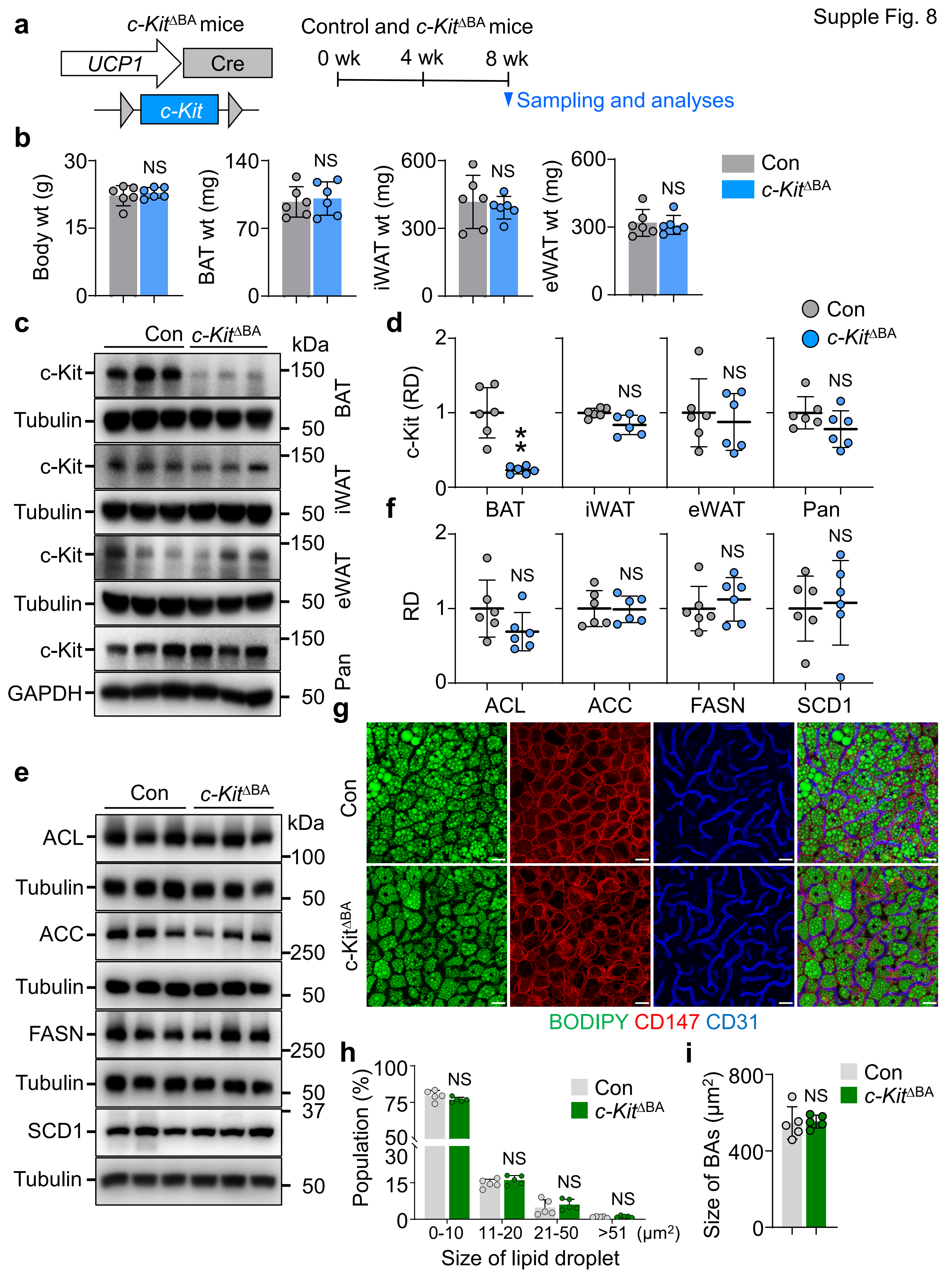
Supple Fig. 8
a
c-Kit$^{\Delta BA}$ mice
UCP1
Cre
c-Kit
Control and c-Kit$^{\Delta BA}$ mice
0 wk
4 wk
8 wk
Sampling and analyses
b
Body wt (g)
BAT wt (mg)
iWAT wt (mg)
eWAT wt (mg)
NS
Con
c-Kit$^{\Delta BA}$
c
Con
c-Kit$^{\Delta BA}$
kDa
c-Kit
Tubulin
GAPDH
150
50
BAT
iWAT
eWAT
Pan
d
c-Kit (RD)
NS
BAT
iWAT
eWAT
Pan
f
RD
ACL
ACC
FASN
SCD1
e
Con
c-Kit$^{\Delta BA}$
kDa
ACL
Tubulin
ACC
FASN
SCD1
100
50
250
37
g
Con
c-Kit$^{\Delta BA}$
BODIPY CD147 CD31
h
Population (%)
NS
Con
c-Kit$^{\Delta BA}$
0-10
11-20
21-50
>51 (μm^2)
Size of lipid droplet
i
Size of BAs (μm^2)
NS
Con
c-Kit$^{\Delta BA}$

Supplementary Fig. 8. BA-specific c-Kit deletion does not alter body weight, weights of adipose tissues, protein levels of lipogenic enzymes, and sizes of adipocytes in the BAT

a, Diagrams for the generation of BA-specific c-Kit deletion (*c-Kit*$^{\Delta BA}$) mouse and analyses of 8-week-old control (Con) and *c-Kit*$^{\Delta BA}$ mice.
b, Comparisons of body weight and fat weights in Con and *c-Kit*$^{\Delta BA}$ mice. Each dot indicates a value from one mouse and n = 6 mice/group from two independent experiments. Vertical bars indicate mean ± SD. NS, not significant *versus* Con by two-tailed t-test.
c,**d**, Representative immunoblotting and comparisons of relative density (RD) of c-Kit in the indicated adipose tissues and pancreas from Con and *c-Kit*$^{\Delta BA}$ mice. The same amount of protein loading in each lane is verified by immunoblotting of tubulin or GAPDH. Each dot indicates a value from one mouse and n = 6 mice/group from two independent experiments. Vertical bars indicate mean ± SD. $^{**}P<0.01$ *versus* Con by two-tailed t-test. NS, not significant. Protein sizes are indicated as kilodalton (kDa).
e,**f**, Representative immunoblotting and comparisons of RD of ACC, ACL, FASN, and SCD1 in BATs from Con and *c-Kit*$^{\Delta BA}$ mice. The same amount of protein loading in each lane is verified by immunoblotting of tubulin. Each dot indicates a value from one mouse and n = 6 mice/group from two independent experiments. Vertical bars indicate mean ± SD. NS, not significant *versus* Con by two-tailed t-test. Protein sizes are indicated as kilodalton (kDa).
g-**i**, Representative images and comparisons of the populations of different sizes of lipid droplets and sizes of $CD147^{+}$ BAs in BATs from Con and *c-Kit*$^{\Delta BA}$ mice. Scale bars, 20 µm. Each dot indicates the population (%) of the total of 1,200-2,400 droplets (100%) from 3 portions of BAT in one mouse and n = 5 mice/group from two independent experiments. Vertical bars indicate mean ± SD. NS, not significant *versus* Con by two-way ANOVA test followed by Sidak test (**h**) and by two-tailed t-test (**i**).

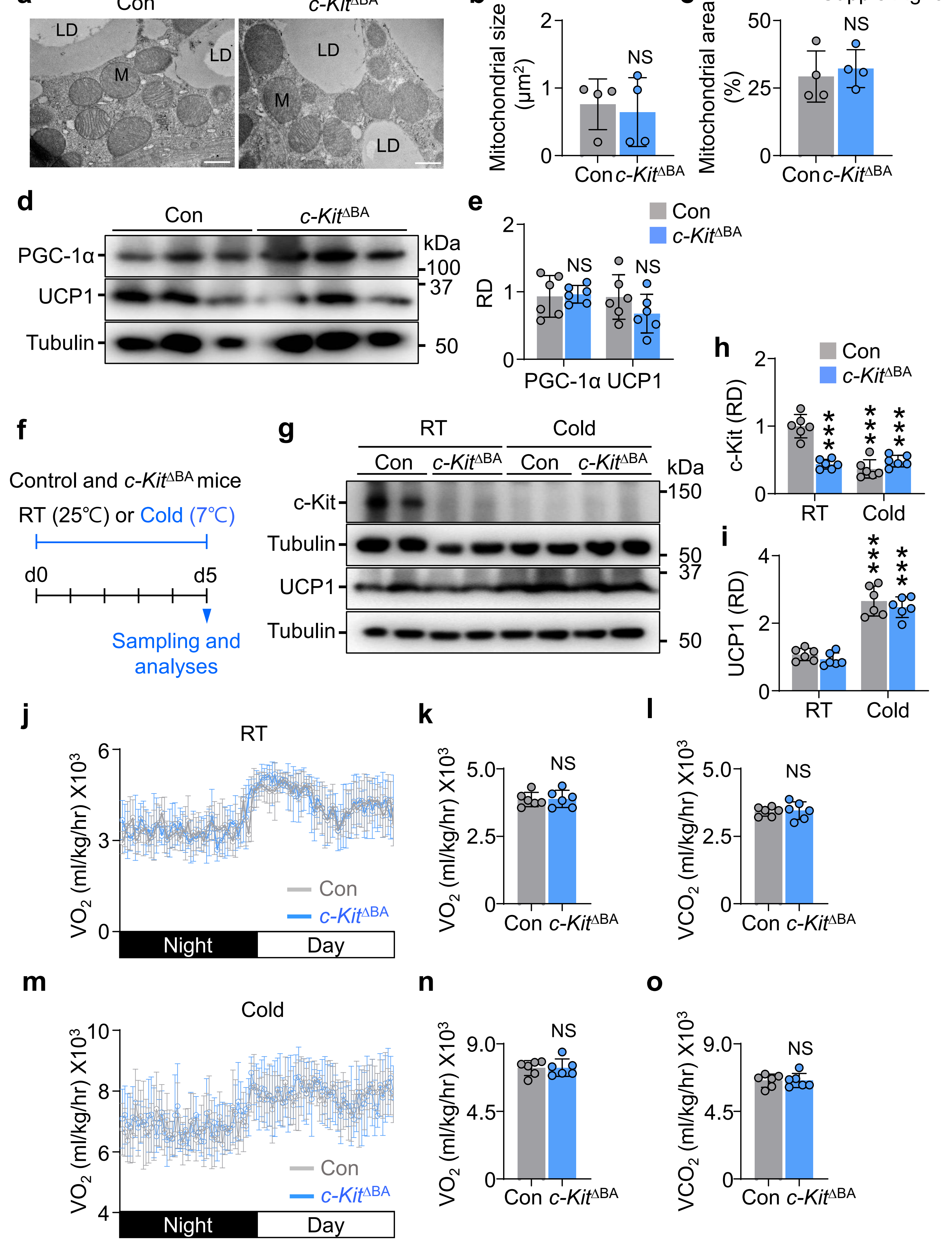
a
Con
c-Kit$^{\Delta BA}$
LD
M
b
Mitochondrial size (μm^2)
NS
c
Mitochondrial area (%)
NS
d
PGC-1α
UCP1
Tubulin
kDa
100
37
50
e
RD
PGC-1α UCP1
f
Control and c-Kit$^{\Delta BA}$ mice
RT (25℃) or Cold (7℃)
d0
d5
Sampling and analyses
g
RT
Cold
c-Kit
150
h
c-Kit (RD)
i
UCP1 (RD)
j
RT
VO_2 (ml/kg/hr) X10^3
Night
Day
k
l
VCO_2 (ml/kg/hr) X10^3
m
Cold
n
o

Supplementary Fig. 9. BA-specific c-Kit deletion mice do not show altered thermogenesis

a-**c**, Representative electron microscopic (EM) images of mitochondria and comparisons of their size and area in BAT of 8-week-old Con *versus* *c-Kit*$^{\Delta BA}$ mice. Scale bars, 600 nm. LD, lipid droplet. M, mitochondria. Dots and bars indicate mean ± SD from n = 4/group from two independent experiments. NS, not significant *versus* Con by two-tailed t-test.
d,**e**, Immunoblotting and comparisons of relative density (RD) of PGC-1α and UCP1 in BAT in Con *versus* *c-Kit*$^{\Delta BA}$ mice at RT. The same amount of protein loading in each lane is verified by immunoblotting of tubulin. Each dot indicates a value from one mouse and n = 6 mice/group from two independent experiments. Vertical bars indicate mean ± SD. NS, not significant *versus* Con by two-tailed t-test. Protein sizes are indicated as kilodalton (kDa).
f, Diagram for the experimental procedure for cold (7℃) exposure for 5 days in 8-week-old Con and *c-Kit*$^{\Delta BA}$ mice~~,~~ and sampling for the analyses.
g-**i**, Immunoblotting and comparisons of relative density (RD) of c-Kit and UCP1 in BATs from Con and *c-Kit*$^{\Delta BA}$ mice at 25℃ or 7℃. The same amount of protein loading in each lane is verified by immunoblotting of tubulin. Each dot indicates a value from one mouse and n = 6 mice/group from two independent experiments. Vertical bars indicate mean ± SD. ****P*<0.001 *versus* Con RT by one-way ANOVA test followed by Tukey's *post-hoc* test. Protein sizes are indicated as kilodalton (kDa).
j-**o**, Oxygen and carbon dioxide consumption of Con and *c-Kit*$^{\Delta BA}$ mice at 25℃ or 7℃. Each dot indicates a value from one mouse and n = 6 mice/group. Vertical bars indicate mean ± SD. NS, not significant *versus* Con by two-tailed t-test.

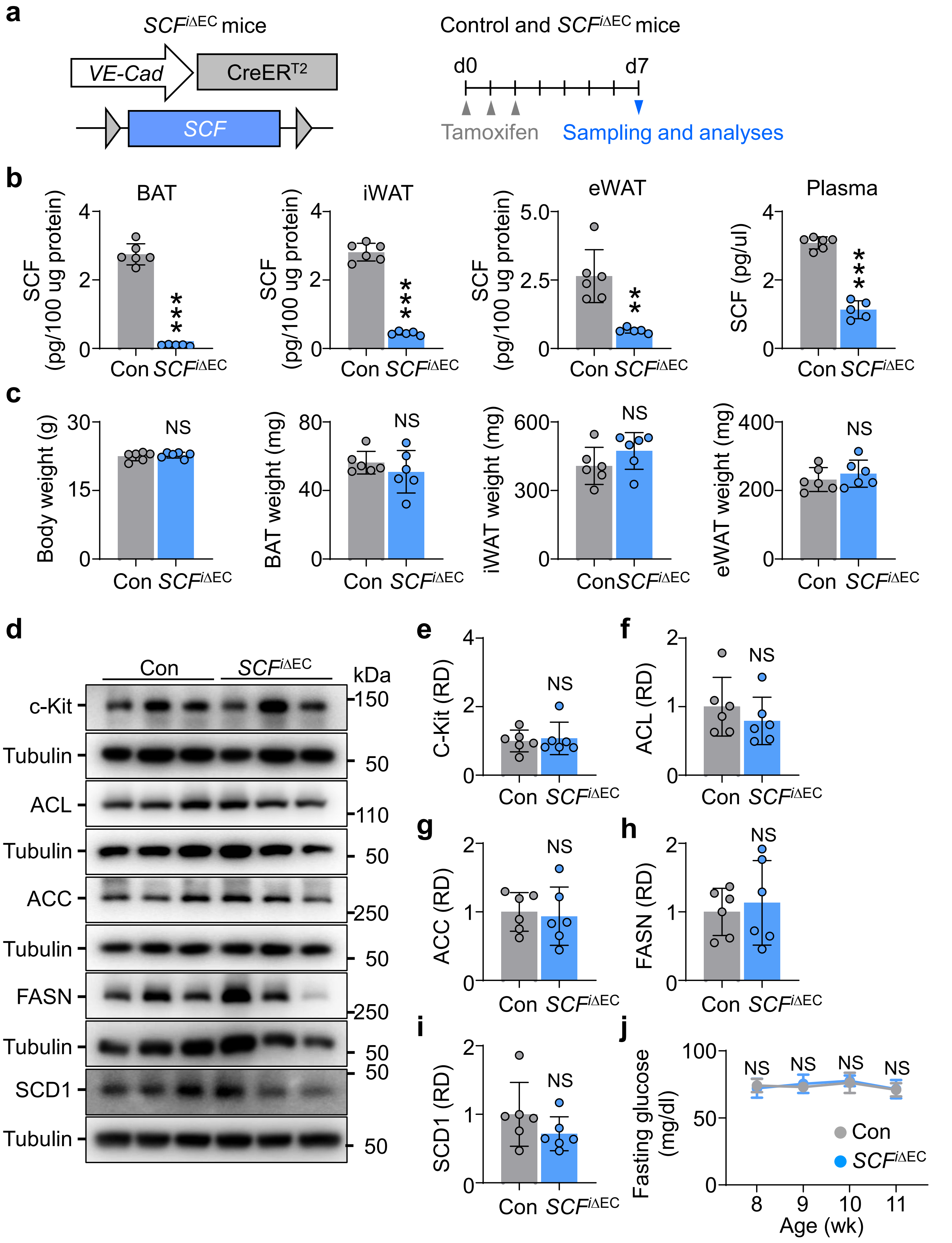
a
SCF$^{i\Delta EC}$ mice
VE-Cad
CreER^{T2}
SCF
Control and SCF$^{i\Delta EC}$ mice
d0
d7
Tamoxifen
Sampling and analyses
b
BAT
iWAT
eWAT
Plasma
SCF (pg/100 ug protein)
SCF (pg/ul)
Con
SCF$^{i\Delta EC}$
c
Body weight (g)
BAT weight (mg)
iWAT weight (mg)
eWAT weight (mg)
NS
d
c-Kit
Tubulin
ACL
ACC
FASN
SCD1
kDa
150
50
110
250
e
C-Kit (RD)
f
ACL (RD)
g
ACC (RD)
h
FASN (RD)
i
SCD1 (RD)
j
Fasting glucose (mg/dl)
Age (wk)
8
9
10
11

Supplementary Fig. 10. EC-specific SCF deletion does not alter body weight, weights of adipose tissues, protein levels of lipogenic enzymes in the BAT, and fasting blood glucose levels

a, Schematic diagram for the generation of inducible EC-specific SCF deletion ($SCF^{i\Delta EC}$) mice, and the experimental procedure for tamoxifen administrations, sampling, and analyses of 8-week-old control (Con) and $SCF^{i\Delta EC}$ mice.
b, Comparisons of SCF concentrations in adipose tissues and plasma from Con and $SCF^{i\Delta EC}$ mice. Each dot indicates a value from one mouse and n = 5-6 mice/group from two independent experiments. Vertical bars indicate mean ± SD. $^{**}P<0.01$, $^{***}<0.001$ *versus* Con by two-tailed t-test.
c, Comparisons of body weight and adipose tissue weights of Con and $SCF^{i\Delta EC}$ mice. Each dot indicates a value from one mouse and n = 6 mice/group from two independent experiments. Vertical bars indicate mean ± SD. NS, not significant *versus* Con by two-tailed t-test.
d-**i**, Representative immunoblotting and comparisons of c-Kit, ACL, ACC, FASN, and SCD1 in BATs from Con and $SCF^{i\Delta EC}$ mice. The same amount of protein loading in each lane is verified by immunoblotting of tubulin. Each dot indicates a value from one mouse and n = 6 mice/group from two independent experiments. Vertical bars indicate mean ± SD. NS, not significant *versus* Con by two-tailed t-test. Protein sizes are indicated as kilodalton (kDa).
j, Comparisons of weekly fasting blood glucose levels in Con and $SCF^{i\Delta EC}$ mice during 8-11 weeks of age. Dots and bars indicate mean ± SD from n = 6 mice/group. NS, not significant *versus* Con by two-tailed t-test.

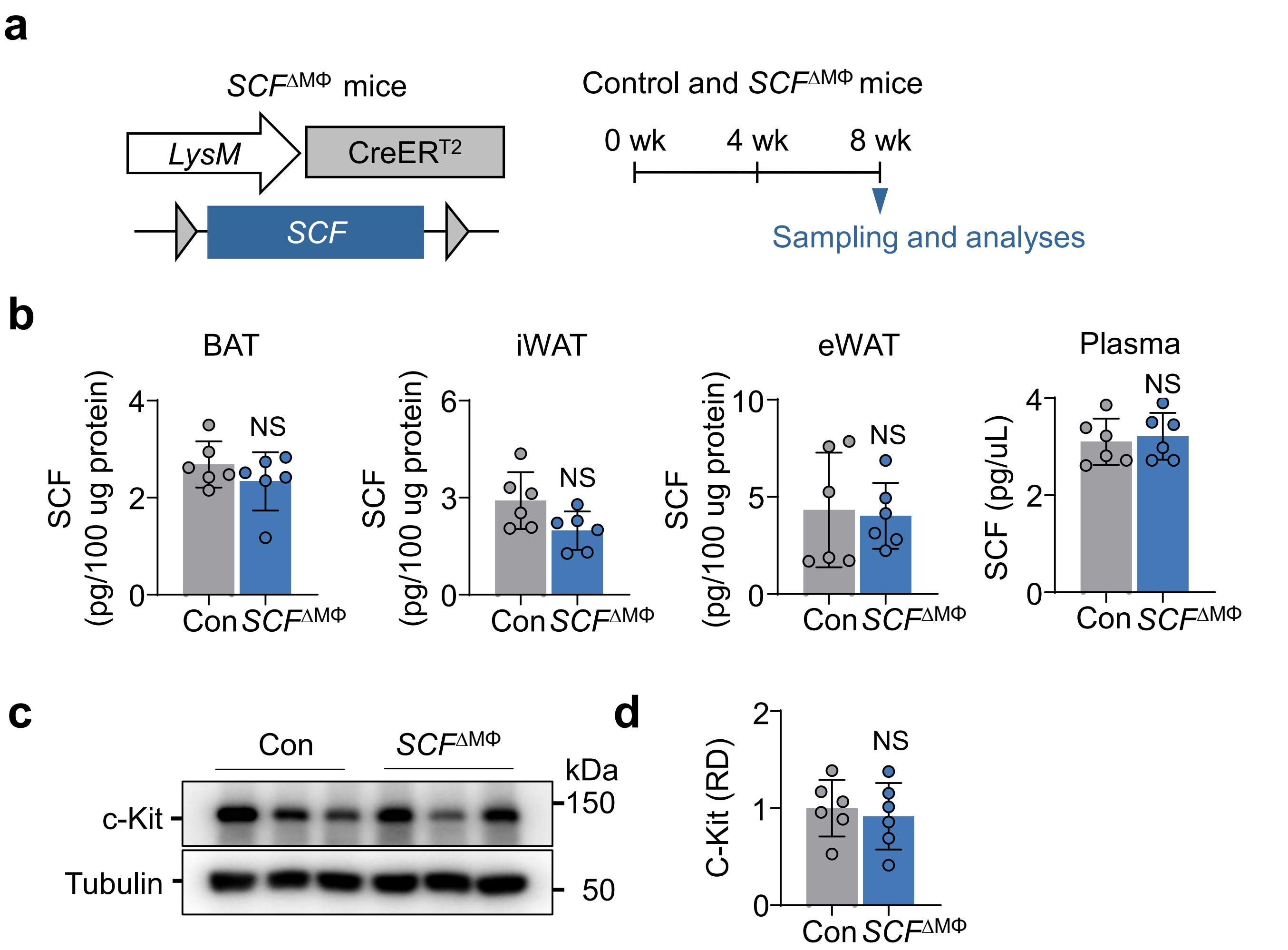

Supplementary Fig. 11. Macrophage-specific SCF deletion neither alters SCF levels in the adipose tissues and plasma nor changes c-Kit in BAT

a, Schematic diagram for generation of macrophage-specific SCF deletion (*SCF*$^{\Delta M\Phi}$) mice and the experimental procedure for sampling and analyses of 8-week-old control (Con) and *SCF*$^{\Delta M\Phi}$ mice.
b, Comparisons of SCF concentrations in the adipose tissues and plasma of Con and *SCF*$^{\Delta M\Phi}$ mice. Each dot indicates a value from one mouse and n = 6 mice/group from two independent experiments. Vertical bars indicate mean ± SD. NS, not significant *versus* Con by two-tailed t-test.
c,**d**, Representative immunoblotting and comparisons of c-Kit in BAT from Con and *SCF*$^{\Delta M\Phi}$ mice. The same amount of protein loading in each lane is verified by immunoblotting of tubulin. Each dot indicates a value from one mouse and n = 6 mice/group from two independent experiments. Vertical bars indicate mean ± SD. NS, not significant *versus* Con by two-tailed t-test. Protein sizes are indicated as kilodalton (kDa).

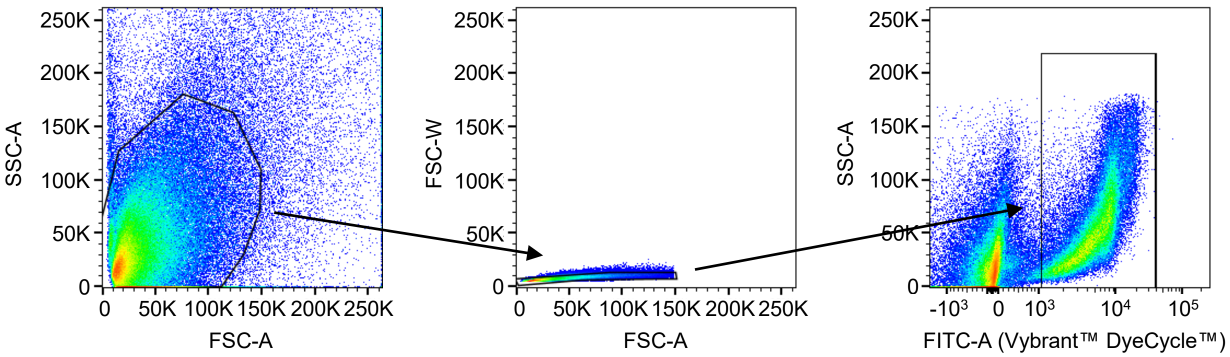

Supplementary Fig. 12. FASC gating strategy for isolating nuclei from ECs, PCs, BAs, and WAs in the BAT of adult C57BL/6J mice. In the total nuclei population defined by SSC-A and FSC-A detector, doublet nuclei were removed by the gating in FSC-W and FSC-A detector, and then only the Vybrant™ DyeCycle™-positive nuclei were isolated by a FACS.
