# Supplementary material for: Endothelial cell-derived stem cell factor promotes lipid accumulation through c-Kit-mediated increase of lipogenic enzymes in brown adipocytes
Source: Nat Commun. 2023 May 13;14:2754. doi: 10.1038/s41467-023-38433-5 (PMC10183046; doi:10.1038/s41467-023-38433-5)
Supplement: Supplementary file 3 — Reporting Summary [file 41467_2023_38433_MOESM3_ESM.pdf]

nature portfolio

Corresponding author(s): Gou Young Koh
Last updated by author(s): Apr 24, 2023

Reporting Summary

Nature Portfolio wishes to improve the reproducibility of the work that we publish. This form provides structure for consistency and transparency in reporting. For further information on Nature Portfolio policies, see our Editorial Policies and the Editorial Policy Checklist.

Statistics

For all statistical analyses, confirm that the following items are present in the figure legend, table legend, main text, or Methods section.

n/a	Confirmed	
☐	☒	The exact sample size (n) for each experimental group/condition, given as a discrete number and unit of measurement
☐	☒	A statement on whether measurements were taken from distinct samples or whether the same sample was measured repeatedly
☐	☒	The statistical test(s) used AND whether they are one- or two-sided<br>*Only common tests should be described solely by name; describe more complex techniques in the Methods section.*
☐	☒	A description of all covariates tested
☐	☒	A description of any assumptions or corrections, such as tests of normality and adjustment for multiple comparisons
☐	☒	A full description of the statistical parameters including central tendency (e.g. means) or other basic estimates (e.g. regression coefficient) AND variation (e.g. standard deviation) or associated estimates of uncertainty (e.g. confidence intervals)
☐	☒	For null hypothesis testing, the test statistic (e.g. F, t, r) with confidence intervals, effect sizes, degrees of freedom and P value noted<br>*Give P values as exact values whenever suitable.*
☒	☐	For Bayesian analysis, information on the choice of priors and Markov chain Monte Carlo settings
☒	☐	For hierarchical and complex designs, identification of the appropriate level for tests and full reporting of outcomes
☒	☐	Estimates of effect sizes (e.g. Cohen's d, Pearson's r), indicating how they were calculated

Our web collection on statistics for biologists contains articles on many of the points above.

Software and code

Policy information about availability of computer code

Data collection	Amersham Imager 600 (GE Healthcare Life Sciences)<br>Transmission electron microscope (FEI, Tecnai G2 spirit TWIN)<br>LSM880 confocal microscope (Carl Zeiss)<br>Real-time PCR system (Applied Biosystems, Waltham, MA, USA)<br>Oxymax/CLAMS calorimetry system (Columbus Instruments)<br>Waters Xevo G2-XS QTOF MS (Waters Corp., Milford, MA, USA)
Data analysis	Zen 2.3 software (Carl Zeiss)<br>Image Studio Lite (LI-COR)<br>GraphPad Prism 7.0 (GraphPad Software)<br>ImageJ (version 1.8.0_172) software (NIH)<br>R: The R Project for Statistical Computing (version 4.0.3)<br>R: package 'Seurat' (version 4.0.6)<br>NanoSprint12 CMOS camera (Advanced Microscopy Techniques)<br>Progenesis software (Waters)

For manuscripts utilizing custom algorithms or software that are central to the research but not yet described in published literature, software must be made available to editors and reviewers. We strongly encourage code deposition in a community repository (e.g. GitHub). See the Nature Portfolio guidelines for submitting code & software for further information.

Data

Policy information about availability of data

All manuscripts must include a data availability statement. This statement should provide the following information, where applicable:

- Accession codes, unique identifiers, or web links for publicly available datasets
- A description of any restrictions on data availability
- For clinical datasets or third party data, please ensure that the statement adheres to our policy

Single-cell RNA sequencing data are available in National Center for Biotechnology Information's Gene Expression Omnibus under accession number GSE207096 (https://www.ncbi.nlm.nih.gov/geo/query/acc.cgi?acc=GSE207096). The remaining data are available within the Article or Additional Information. Source data are provided as a Source Data file. Further information and requests for resources and reagents should be directed to and will be fulfilled by Hyuek Jong Lee (hyuekjong.lee@gmail.com) and Gou Young Koh (gykoh@kaist.ac.kr).

Human research participants

Policy information about studies involving human research participants and Sex and Gender in Research.

Reporting on sex and gender	N/A
Population characteristics	N/A
Recruitment	N/A
Ethics oversight	N/A

Note that full information on the approval of the study protocol must also be provided in the manuscript.

Field-specific reporting

Please select the one below that is the best fit for your research. If you are not sure, read the appropriate sections before making your selection.

☒ Life sciences ☐ Behavioural & social sciences ☐ Ecological, evolutionary & environmental sciences

For a reference copy of the document with all sections, see nature.com/documents/nr-reporting-summary-flat.pdf

Life sciences study design

All studies must disclose on these points even when the disclosure is negative.

Sample size	Sample sizes were chosen on the basis of standard power calculations (with α = 0.05 and power of 0.8) performed for similar experiments and statistical methods were not used to predetermine sample sizes as previously published (Robciuc et al., Cell Metabolism, 2016; Louveau et al., Nature, 2015).
Data exclusions	No samples were excluded from the analysis.
Replication	Experiments were replicated at least once for all analyses to produce convincing results and the number of reproductions of each experimental finding is described in each figure legends. All attempts at experimental replication were successful.
Randomization	Because the experiments required a specific genetic signature, randomization per treatment was not possible. The animal studies were performed using littermate controls. For the experiments of snRNA-seq, cold exposure, CL316,247 treatment, UDN, or thermoneutrality exposure, C57BL/6J mice were randomly chosen.
Blinding	The investigators were blinded during the experiments and the result analyses.

Reporting for specific materials, systems and methods

We require information from authors about some types of materials, experimental systems and methods used in many studies. Here, indicate whether each material, system or method listed is relevant to your study. If you are not sure if a list item applies to your research, read the appropriate section before selecting a response.

Materials & experimental systems

n/a	Involved in the study
	☒ Antibodies
☒	☐ Eukaryotic cell lines
☒	☐ Palaeontology and archaeology
	☒ Animals and other organisms
☒	☐ Clinical data
☒	☐ Dual use research of concern

Methods

n/a	Involved in the study
☒	☐ ChIP-seq
	☒ Flow cytometry
☒	☐ MRI-based neuroimaging

Antibodies

Antibodies used

The following primary and secondary antibodies were used in the immunostaining: anti-CD31 (1:1000, hamster monoclonal, #MAB1398Z, clone TLD-3A12, Millipore); anti-GFP (1:1000, goat polyclonal, #ab6658, Abcam); anti-F4/80 (1;1000, rat monoclonal, #MCA497GA, clone Cl:A3-1, Bio-Rad); Alexa FluorTM 647 conjugated anti-phalloidin [1:1000, mouse, #8940, Cell Signaling Technology (CST)]; PE-conjugated CD147 (1:200, rat monoclonal, #123707, clone
OX-114, BioLegend); and Cy3-, Cy5-, or Alexa488-conjugated, anti-rat, anti-goat or anti-hamster secondary antibodies (diluted at a a ratio of 1:1000, #112-585-167, #127-605-160, #127-165-160, #127-545-160, #705-546-147) were purchased from Jackson ImmunoResearch. The following primary and secondary antibodies were used in the immunoblotting; rabbit anti-c-Kit (1:5000, D13A, #3074); rabbit anti-ACC (1:5000, C83B10, #3676); rabbit anti-FASN (1:5000, C20G5, #3180); rabbit anti-SCD1 (1:5000, C12H5, #2794); rabbit anti-GAPDH (1:5000, D16H11, #5174) (CST); rabbit anti-p-AKT1 (1:5000, ser473, D7F10, #9018); rabbit-anti-AKT1 (1:5000, C73H10, #2938); rabbit-anti-p-AKT2 (Ser474) (1:5000, D3H2, #8599); rabbi-anti-AKT2 (1:5000, D6G4, #3063); rabbi-anti-p-p44/42 MAPK (Thr202/Tyr204) (1:5000, 197G2, #4377); rabbi-anti-p44/42 MAPK (1:5000, 137F5, #4695); rabbit anti-PGC1-α (1:5000, #ab191838); rabbit anti-tyrosine hydroxylase (1:5000, EP1532Y, #ab137869); rabbit anti-ACL (1:5000, EP704Y, #ab40793); rabbit anti-UCP1 (1:5000, #ab23841) (Abcam) (4 h at RT or overnight); rat anti-α-tubulin (1:5000, 3H3085, #sc-69970, Santacruz); and anti-rabbit (1:5000, #7074, CST) or anti-mouse (1:5000, #7076, CST) secondary peroxidase-conjugated antibodies. Neutral lipids in adipocytes were stained with BODIPYTM 493/503 (#D3922, Invitrogen) or lipidTOX red dyes (1:1000, #H34476, Invitrogen).

Validation

All the antibodies were validated for the species (mouse) and applications (immunohistochemistry and immunoblotting) by the correspondent manufacturer, which is described in the manufacturer's website. Our usage was described in the Methods section of the manuscript as below.
For immunofluorescence staining (IFS), the tissues were permeabilized and blocked with blocking buffer containing 5% donkey serum in 1% Triton-X 100 in PBS for 1 h at room temperature (RT). Then, they were incubated with a primary antibody diluted in the blocking buffer overnight at 4°C. After several washes with PBS, they were incubated with secondary antibodies (Jackson ImmunoResearch) diluted in the blocking buffer for 4 h at RT. After several washes with PBS, they were mounted with Vecta-shield (Vector Laboratories).
For immunoblotting, membranes were incubated with 2% bovine serum albumin (BSA) blocking buffer for 1 h at RT, and blotted with the following antibodies overnight at 4°C. After several washes with TBST, they were incubated with secondary antibodies (Cell Signaling Technology) for 1 h at RT. Target proteins were detected using ECL western blot detection solution (#WBKLS0500, Millipore). The same amount of protein loading in each lane is verified by immunoblotting of tubulin or GAPDH.

Animals and other research organisms

Policy information about studies involving animals; ARRIVE guidelines recommended for reporting animal research, and Sex and Gender in Research

Laboratory animals

Scf+/gfp and Scf flox/flox mice (Ding et al., Nature, 2012 and Shen et al., Nature, 2021) were provided by Dr. Sean Morrison (Southwestern University, USA); c-Kit-CreERT2 mice (He et al., Nat Med, 2017) were provided by Dr. Bin Zhou (Chinese Academy of Sciences in Shanghai, China); VE-cadherin-Cre-ERT2 mice (Okabe, K. et al., Cell, 2014) were provided by Dr. Yoshiaki Kubota (Keio University); C57BL/6J, tdTomatoflox/flox (#007909), c-Kit flox/flox (#042035)64, UCP1-Cre (#024670), and LysM-Cre (#004781) mice were obtained from The Jackson Laboratory. All mice were bred and maintained under specific pathogen-free conditions at KAIST. Mice were housed under a 12 h light/dark cycle within a temperature-controlled room (21–22°C) and allowed to free access food (Teklad global 18% protein rodent diet, #2018C, Envigo®) and water

Wild animals

The study dose not involve wild animals.

Reporting on sex

To avoid the off-target effect of tamoxifen on iWAT browning in the CreERT2 female mice (Zhao L et al., Int. J. Obes, 44:226-234, 2020) and to preclude the possible sexual differences among the mice groups, only male mice were used in all of this study.

Field-collected samples

The study does not involve samples collected from the field.

Ethics oversight

Animal care and experimental procedures were performed under the approval (KA2018-70) of the Institutional Animal Care and Use Committee of Korea Advanced Institute of Science and Technology (KAIST).

Note that full information on the approval of the study protocol must also be provided in the manuscript.

Flow Cytometry

Plots

Confirm that:

☒ The axis labels state the marker and fluorochrome used (e.g. CD4-FITC).

☒ The axis scales are clearly visible. Include numbers along axes only for bottom left plot of group (a 'group' is an analysis of identical markers).

☒ All plots are contour plots with outliers or pseudocolor plots.

☒ A numerical value for number of cells or percentage (with statistics) is provided.

Methodology

Sample preparation	Interscapular BATs were harvested from 8-week-old C57BL/6J mice and incubated in RPMI buffer containing 0.1% collagenase type II (#LS004177, Worthington), 0.1% dispase (#17105041, Gibco), and 0.05% trypsin (#T4799, Sigma-Aldrich) for 60 min at 37°C with constant shaking at 1,000 rpm (Eppendorf thermomixer, Sigma-Aldrich). After inactivating collagenase with RPMI buffer containing 10% fetal bovine serum (FBS) (#26140079, Gibco), the cell suspension was filtered through a 100μm nylon mesh (BD Biosciences), followed by centrifugation at 1,000 rpm for 5 min. Because massive amounts of fragmented vasculatures (FVs) were already aggregated with floating BAs after centrifugation, the entire floating cells were digested in Nuclei EZ lysis buffer (#NUC-101, Sigma-Aldrich) to disrupt cell membranes for the isolation of nuclei. The digestion was filtered through a 40μm nylon mesh, followed by centrifugation at 2,000 rpm for 10 min. The resulting pellet was incubated with Vybrant™ DyeCycle™ (#V35004, Thermo Fisher Scientific) for 30 min at 4°C to identify the nuclei
Instrument	BD FACS Aria cell sorter (BD Biosciences)
Software	Diva software (V 8.0)
Cell population abundance	After sorting nuclei, Vybrant™ DyeCycle™ positive nuclei were confirmed under an LSM880 confocal microscope (Carl Zeiss).
Gating strategy	In the total nuclei population detected by SSC-A and FSC-A detectors, doublet nuclei were removed by the gating in FSC-W and FSC-A detectors, and then the Vybrant™ DyeCycle™-positive nuclei were sorted (Extended Data Fig. 12). Vybrant™ DyeCycle™-negative cluster was defined as unstained nuclei. Compensation was performed at the time of acquisition in Diva software using compensation beads (#552843, BD Biosciences).

☒ Tick this box to confirm that a figure exemplifying the gating strategy is provided in the Supplementary Information.
